# Elevation of the tumor marker CA19‐9 in a pancreatic cancer survivor with benign prostatic hyperplasia: A clinical case report

**DOI:** 10.1002/ccr3.8929

**Published:** 2024-05-24

**Authors:** Steve D. Pendry, Nimit Singhal, Eu‐Ling Neo, Darren Foreman, Jean M. Winter

**Affiliations:** ^1^ Flinders Health and Medical Research College of Medicine and Public Health Flinders University Bedford Park South Australia Australia; ^2^ Cancer Centre, Royal Adelaide Hospital and School of Medicine The University of Adelaide Adelaide South Australia Australia; ^3^ Hepatopancreatobiliary Surgical Unit Flinders Medical Centre Bedford Park South Australia Australia; ^4^ Hepatobiliary Surgical Unit Royal Adelaide Hospital Adelaide South Australia Australia; ^5^ College of Medicine and Public Health Flinders University Bedford Park South Australia Australia

**Keywords:** carbohydrate antigen 19‐9, pancreatic ductal adenocarcinoma, prostate, prostate specific antigen

## Abstract

Serum carbohydrate antigen 19‐9 (CA19‐9) is used for recurrence surveillance in patients with resected pancreatic ductal adenocarcinoma (PDAC). This report describes the association of increasing CA19‐9 in a male PDAC survivor with presence of prostatic hyperplasia. Unexplained elevation of CA19‐9 in male PDAC survivors might be attributable to benign prostatic conditions.

## 1 INTRODUCTION

Pancreatic ductal adenocarcinoma (PDAC) is the most common type of pancreatic cancer, accounting for over 90% of cases, and arises from the epithelial cells lining the pancreatic duct. In 2020, PDAC was the 7th leading cause of cancer‐related death, with almost half a million people worldwide being diagnosed with PDAC and an equivalent number of people also losing their life to this disease.^1^ Although a relatively uncommon cancer compared to other solid tumors such as breast and prostate cancer, PDAC has only a ~10% five‐year overall survival rate and a median overall survival time of ~7 months.^2^ Due to a lack of reduction in incidence and mortality rates for PDAC compared to other common solid malignancies in recent years, it is estimated to become the second and third leading cause of cancer‐related deaths in the United States and Europe by the years 2030 and 2025, respectively.^3^ There are currently no available screening test for PDAC, and the majority of patients with early‐stage PDAC are asymptomatic. Consequently, most PDAC patients present at an advanced stage. However, patients who undergo successful surgical resection of localized disease can have significantly improved outcomes, where the 5‐year survival is approximately 50%.^4^ It is therefore important to improve early detection, optimize treatment strategies, and improve surveillance techniques for disease recurrence of PDAC in order to reduce PDAC‐associated mortality.

There are currently no approved blood tests or screening programs for detection of PDAC. It is usually only identified by radiological scans inadvertently or after the patient experiences symptoms, at which point the cancer is usually already advanced. The carbohydrate antigen 19‐9 (CA19‐9) is regularly measured in the blood from patients with pancreatic cancer. However, CA19‐9 is limited to monitoring for disease recurrence and in some cases, monitoring treatment efficacy.^5^ CA19‐9 is not useful as a general population screening test as it is not cancer‐specific. It can be elevated in other common conditions and some individuals whose CA19‐9 levels do not change even in the presence of malignancy.^6^ Furthermore, some environmental exposures can lead to elevated CA19‐9 including excessive black tea consumption.^7^ Since CA19‐9 may be elevated in other scenarios, it is important for clinicians to be aware of what indications may lead to false‐positive CA19‐9 blood tests in PDAC survivors. This is important to reduce any associated stresses of any unexplained increase in CA19‐9 readings on the patient, reduce excessive radiation exposure with unnecessary radiological scans, and more importantly, direct the patient to appropriate referral and follow‐up to other specialties in a timely manner.

This review presents the result of an investigation into a pancreatic cancer patient in remission with unexplained rising CA19‐9 serum levels and its association with prostate‐specific antigen biomarker levels and presence of benign prostatic hyperplasia.

## 2 METHODS

The patient was diagnosed with stage IIA borderline resectable pancreatic adenocarcinoma in December 2013 at the age of 65 years. The tumor was located in the uncinate process of pancreas measuring 28 × 18 mm, abutting the superior mesenteric vein (SMV). The SMV contained a tumor thrombus extending for a length of 75 mm inferiorly into its tributaries. The patient's CA19‐9 serum levels at diagnosis were 122 U/mL (reference level 37 U/mL). Three months of aggressive neo‐adjuvant chemotherapy (modified FOLFIRINOX) was prescribed to downstage the tumor, and subcutaneous Clexane injections were administered to dissolve the blood clot in the major mesenteric vein. Re‐staging CT scans showed a reduction in the size of the pancreatic primary tumor to 19 × 16 mm, and the SMV had become relatively collapsed (previously expansile with tumor thrombus) suggesting post‐thrombotic stricture. There was no evidence of metastatic disease. Serum CA19‐9 levels measured before treatment were still above reference levels at 264 U/mL (Dec 2013) and 142 U/mL (Mar 2014). The patient subsequently underwent a pancreaticoduodenectomy (Whipple procedure) in late March 2014.

During surgery, the pancreatic tumor was found to be adherent to the SMV however, it was successfully dissected away without requiring a major venous resection. Surgery was followed by 6 weeks of adjuvant radiotherapy coupled with concurrent continuous chemotherapy (5‐fluouracil). On completion of the radiotherapy treatment, adjuvant chemotherapy (gemcitabine) was continued until December 2014.

In March 2015, the patient entered a surveillance/monitoring phase that involved regular measurements of his serum CA19‐9 level, less frequent measurements of carcinoembryonic antigen (CEA) levels plus CT scans every 6 months for the first 2 years followed by annual CT scans. One PET scan was performed in August 2019, which showed no evidence of cancer. Regular electrolytes and liver function tests (ELFT) were also performed. After approximately 2 years (April 2017) post‐adjuvant chemotherapy the patient was able to cease use of pancreatic enzyme supplement with no adverse effects, and his fasting blood glucose concentration has been stable at approximately 5.6 mmol/L.

**FIGURE 1:**
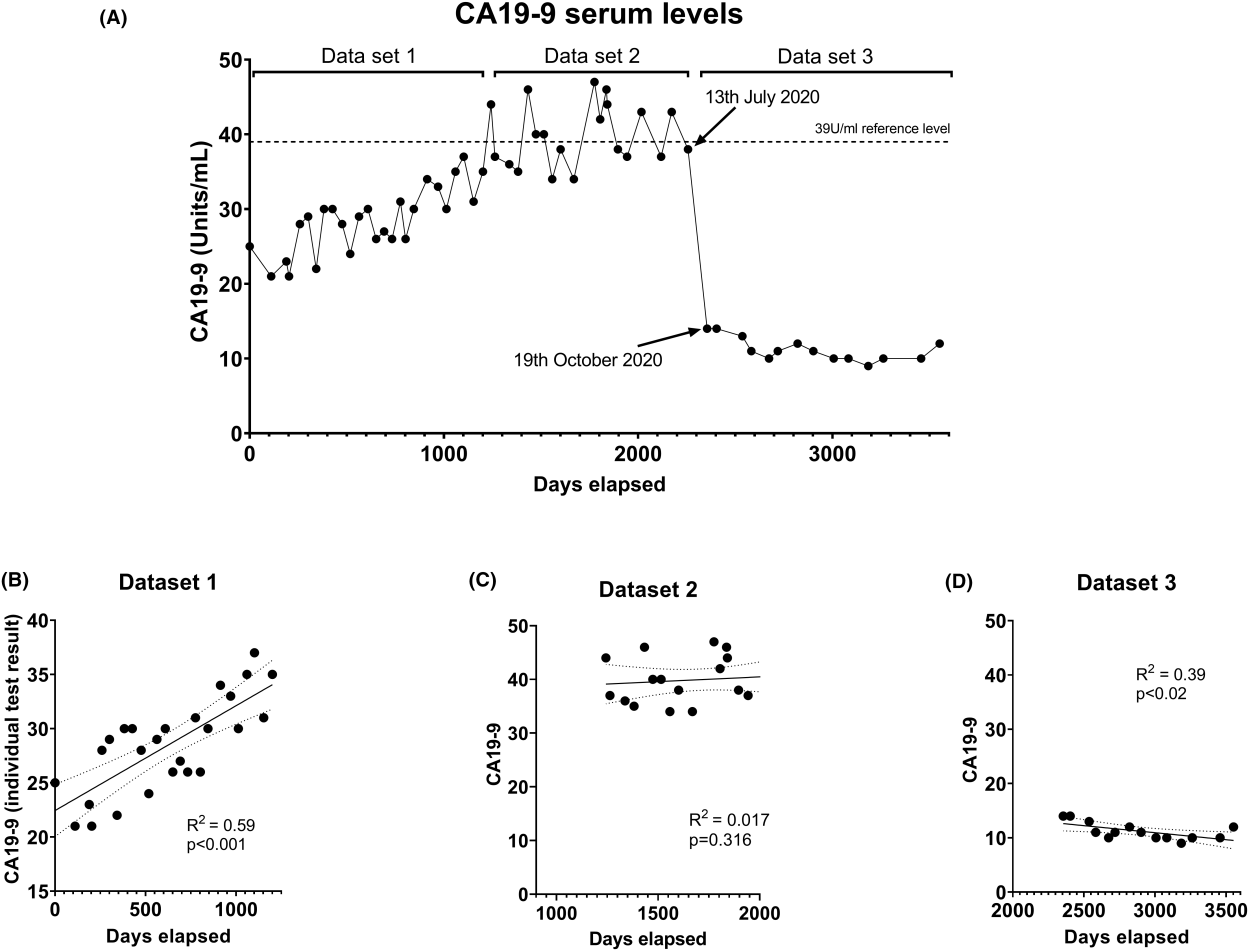
Serum CA19‐9 levels post adjuvant chemoradiotherapy. (A) CA19‐9 readings from May 2014 until October 2023. (B) Simple linear regression of Dataset 1, CA19‐9 readings prior to reaching the reference threshold of 39 U/mL. (C) Simple linear regression of Dataset 2, CA19‐9 levels after reaching the reference threshold but before the significant drop. (D) Simple linear regression of Dataset 3, CA19‐9 readings after the significant drop below the reference threshold. Solid line = line of fit; dotted lines = 95% confidence intervals.

A record of all the patient's CA19‐9 test results from May 2014 to January 2024 is presented in Figure 1A, and yearly average readings are in Table 1. The data reveal a gradual and moderately linear increase (*R* ^2^ = 0.59, *p* < 0.001; Figure 1B) in the mean CA19‐9 level from approximately 25 U/mL to a mean level of 40 U/mL (reference level is 39 U/mL) over a period of approximately 2.5 years. No cause for this steady increase in CA19‐9 readings could be determined, and scans revealed no evidence of cancer. There were hypotheses that the CA19‐9 increases might have been due to the slow thickening of surgical scar tissue from the Whipple procedure or other surgical‐related issues.^8^

**TABLE 1:** Yearly CA19‐9 average readings.

Year	Average reading (U/mL)
2014–2015	23.6
2015–2016	27.5
2016–2017	30.0
2017–2018	37.2
2018–2019	40.4
2019–2020	35.3
2020–2021	12.0
2021–2022	10.8
2022–2023	9.7
2023–2024	11.0

The patient's mean CA19‐9 level then stabilized and remained stable at or above the reference level for approximately 2.7 years (*R* ^2^ = 0.017, *p* = 0.316; Figure 1C). In July 2020, the patient's mean CA19‐9 readings suddenly dropped by over 60%, from 38 U/mL to 14 U/mL, 3 months later in October 2020 (Figure 1A). Following this, it then steadily declined for 3 years (*R* ^2^ = 0.39, *p* < 0.02; Figure 1D), with levels well below the reference limit of between 9 and 10 U/mL for approximately 1.5 years.

**FIGURE 2:**
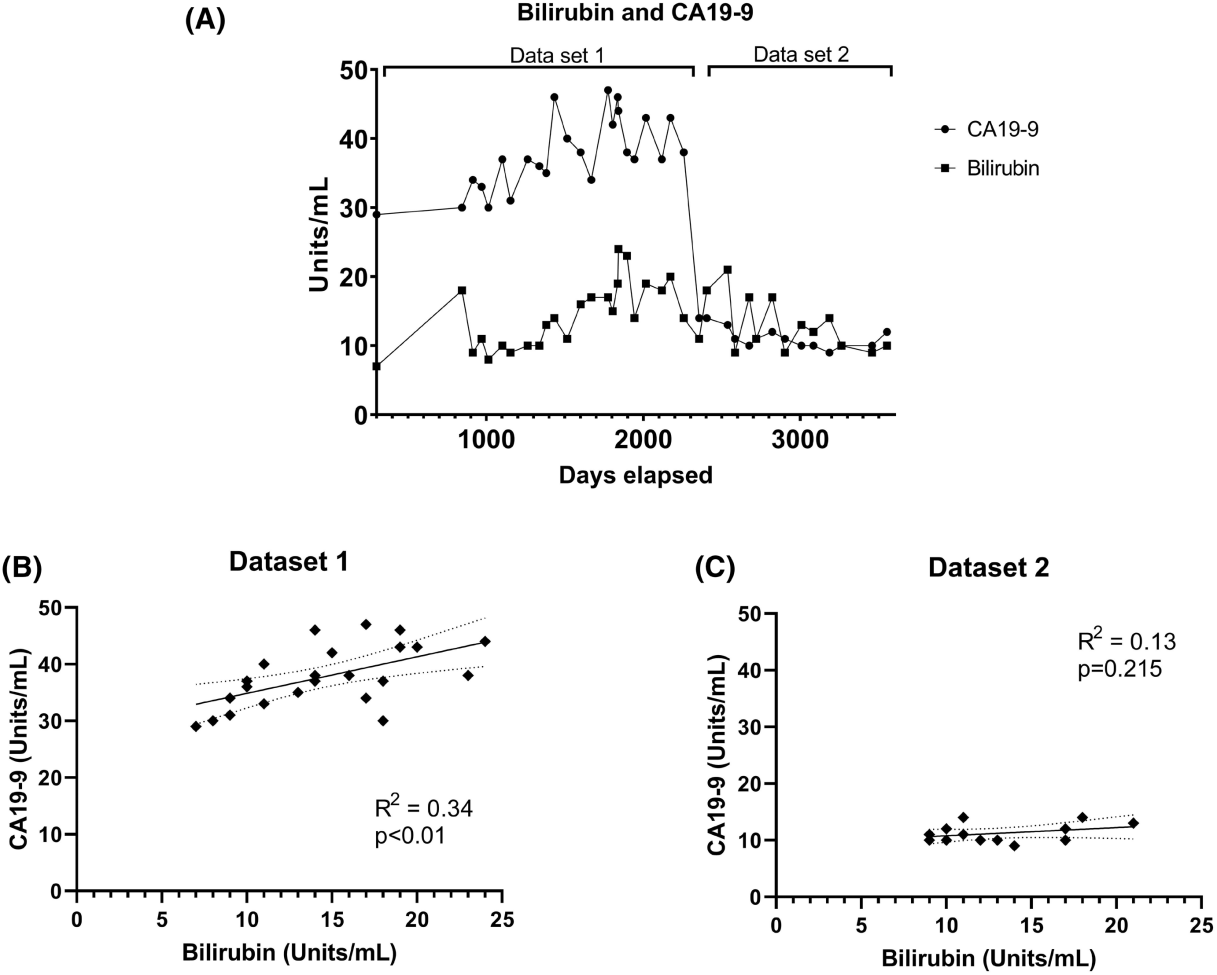
CA19‐9 vs Bilirubin Readings: (A) CA19‐9 and Bilirubin readings overtime from March 2016 until October 2023. (B) Simple linear regression of dataset 1 for CA19‐9 vs bilirubin. (C) Simple linear regression of dataset 2 for CA19‐9 and bilirubin. Solid line = line of fit. Dotted line = 95% confidence intervals.

There is some evidence that CA19‐9 can be attributed to peptic ulcers^9^ or biliary obstruction,^6^, ^10^, ^11^ and so we investigated any association between bilirubin and CA19‐9 levels. There was a modest but significant correlation of bilirubin levels and CA19‐9 prior to the reduction in CA19‐9 levels (*R* ^2^ = 0.335; *p* < 0.01; Figure 2B). However, the bilirubin readings remained relatively stable over the entire surveillance period (*R* ^2^ = 0.0171, *p* = 0.441; Figure 2A), as well as after the CA19‐9 levels dropped 60% (*R* ^2^ = 0.13, *p* = 0.215; Figure 2C), and CT scans during this period revealed no biliary duct obstruction. Similarly, CEA readings taken 1–3 times per year remained well within normal limits of <1.7 μg/L (Table 2).

**TABLE 2:** CEA readings.

Date	CEA (μg/L)
April 3, 2017	0.7
May 22, 2018	1.0
March 19, 2019	1.0
September 03, 2019	<1.7
June 04, 2021	<1.7
October 18, 2021	<1.7
April 19, 2022	<1.7
October 17, 2022	<1.7
April 14, 2022	<1.7
October 26, 2023	<1.7
January 29, 2024	<1.7

## 3 CONCLUSIONS AND RESULTS

In May 2017, the patient's serum prostate‐specific antigen (PSA) was 5.2, having increased from 2.9 in 2013. An MRI of the prostate was arranged, which showed prostatomegaly with a prostate volume of 107 cc and no high‐grade lesions within the prostate. His PSA decreased to 4 ng/mL in January 2018 and rose to 6.5 ng/mL in July 2018, which lead to a prostate biopsy. This confirmed benign prostatic hyperplasia. The patient noticed a deterioration in his urinary stream and had no improvement with an alpha blocker, tamsulosin. Renal ultrasound in June 2020 confirmed that the bladder was emptying poorly with a postvoid residual of 59 mL, and he underwent a transurethral resection of the prostate (TURP) procedure in August 2020. Histopathology confirmed that 66 grams of benign prostate tissue were removed. Following TURP, there was an immediate and significant drop in CA19‐9 levels in October 2020. The patient's oncologist and hepatobiliary surgeon were unaware of the TURP procedure at the time.

**FIGURE 3:**
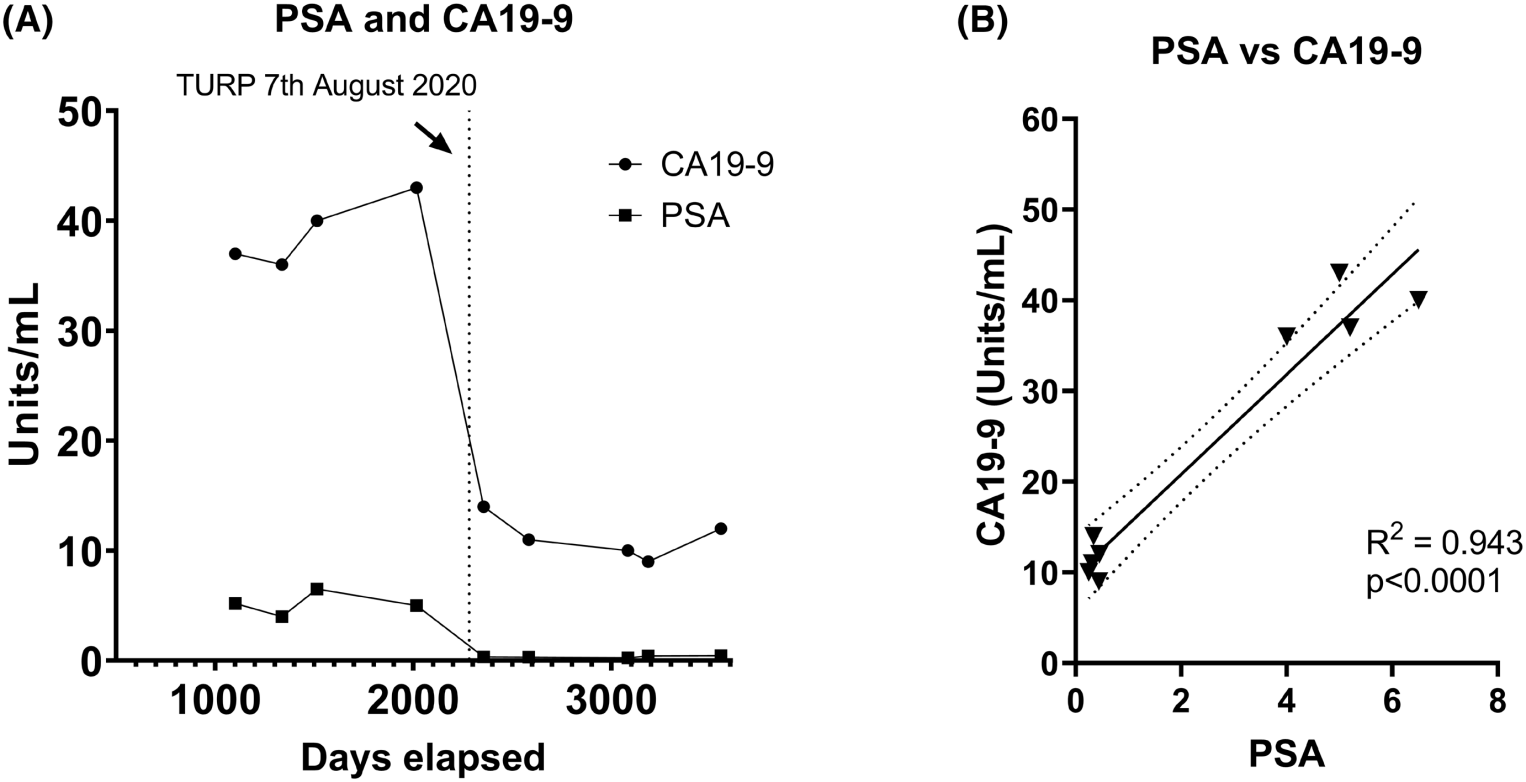
CA19‐9 and PSA serum levels. (A) CA19‐9 and PSA levels from 15th May 2017 to 27th January 2023. (B) Simple linear regression of CA19‐9 and PSA at all‐time points. Solid line = best line of fit. Dotted line = 95% confidence intervals.

A review of the patient's paired PSA and CA19‐9 readings (Figure 3A and Table 3) pre‐ and post‐TURP procedure in August 2020 demonstrates PSA levels were over the reference threshold of 4 ng/mL prior to the TURP followed by a significant 93% reduction of PSA after the TURP, concurrently with the 60% reduction in CA19‐9 levels. When performing a simple linear regression analysis of all PSA and CA19‐9 serum samples, it revealed a highly significant association (*R* ^2^ = 0.938, *p* < 0.0001; Figure 3B). Levels of both PSA and CA19‐9 have since remained stable and well below the reference thresholds for both biomarkers (Figure 3A) and the patient has not had recurrence of their PDAC with the last CT scan in September 2023.

**TABLE 3:** PSA readings.

Date	May 15, 2017	January 5, 2018	July 11, 2018	November 16, 2019	December 7, 2020	June 4, 2021	August 2, 2022	January 27, 2023	January 29, 2024
PSA	5.2	4.0	6.5	5.0	0.34	0.31	0.25	0.44	0.45
CA19‐9	37	36	40	43	14	11	10	9	12

## 4 DISCUSSION

This report investigated the association of CA19‐9 and the prostate cancer biomarker PSA (prostate‐specific antigen) levels pre‐ and post‐transurethral resection of the prostate (TURP) in a 75‐year‐old male PDAC survivor (age of diagnosis 65 y). Longitudinal serum sampling demonstrates a strong and highly significant correlation between PSA and CA19‐9 readings before and after the TURP, which suggests a potential causal link between elevation of the PDAC biomarker CA19‐9 and the presence of benign prostatic hyperplasia (BPH). CA19‐9, also known as sialyl‐Lewis A, is a fucosylated carbohydrate structure produced mainly by epithelial cells across various different organs of the body,^12^ but is particularly high in cells of the gastrointestinal tract and gall bladder.^13^ CA19‐9 is well known to be elevated in pancreatic, upper gastrointestinal tract, and colorectal cancers, as well as other inflammatory conditions of the hepatobiliary system, biliary obstruction and in thyroid diseases.^11^ However, CA19‐9 is not normally expressed in the prostate glandular tissue, and only low levels of expression can be found in the seminal vesicle glandular cells.^13^

Although not common, elevated serum CA19‐9 and increased CA19‐9 tissue expression have been observed in patients with prostate cancer in several case reports,^14^, ^15^, ^16^, ^17^, ^18^, ^19^ but these were mostly in rare prostate cancer subtypes and cases with aggressive metastatic disease. Another case report demonstrated an association between high serum CA19‐9 levels (145.8 U/mL) and presence of a benign cystic tumor of the seminal vesicle, of which the fluid from the cyst itself had a notably high CA19‐9 reading of 4864 U/mL.^20^ To the best of our knowledge, this case report is the first evidence to demonstrate an association of benign prostatic disease and increases in serum CA19‐9. There are several different mechanisms that might explain elevation of CA19‐9 in many different benign diseases. These include response to inflammation and proliferation of normal cells and accumulation of CA19‐9 due to a lack of excretion,^6^ as well as metabolic syndromes,^21^ none of which have been demonstrated to occur in prostatic tissues. Further research into understanding any underlying cause of elevated CA19‐9 expression in prostate tissue is therefore warranted.

Elevated CA19‐9 due to benign prostatic hyperplasia could lead to undue stress and concern for older males who have undergone or are undergoing treatment for PDAC, where elevation of CA19‐9 may lead to suspicions of a return of the cancer. Monitoring serum PSA and CA19‐9 concurrently in older male patients undergoing surveillance for PDAC is therefore justified. Currently, in Australia, the Medicare system limits PSA testing to once annually for men without prostate cancer. PSA testing can be done more frequently at an out‐of‐pocket cost to the patient. More regular PSA testing for pancreatic cancer survivors should be considered in the absence of other, more specific, non‐invasive surveillance testing. Development and implementation of sensitive blood biomarker tests that are more specific for PDAC, such as emerging circulating tumor DNA blood tests,^22^ might improve surveillance strategies and minimize false positive readings caused by conditions other than PDAC.

While this report is based on a single patient study, the strong correlation between CA19‐9 and PSA readings before and after a TURP procedure demonstrates a potential link between benign prostate hyperplasia and increasing CA19‐9 levels. This finding offers clinicians a line of investigation for any male patient who has had, or is being treated for, a cancer for which CA19‐9 is used as a biomarker and is experiencing a slow but significant increase in CA19‐9 readings, which are otherwise not able to be explained or linked directly to the cancer behavior.

## AUTHOR CONTRIBUTIONS

**Steve D. Pendry:** Conceptualization; data curation; formal analysis; investigation; visualization; writing – original draft; writing – review and editing. **Nimit Singhal:** Investigation; methodology; writing – review and editing. **Eu‐Ling Neo:** Investigation; methodology; writing – review and editing. **Darren Foreman:** Investigation; methodology; writing – review and editing. **Jean M. Winter:** Data curation; formal analysis; supervision; visualization; writing – original draft; writing – review and editing.

## FUNDING INFORMATION

Not funded.

## CONFLICT OF INTEREST STATEMENT

The authors have no conflicts of interest to declare.

## CONSENT

Written informed consent was obtained from the patient to publish this report in accordance with the journal's patient consent policy.

## Data Availability

The data that support the findings of this study are available from the corresponding author upon reasonable request.
